# Exploring factors influencing depression among Polish nurses during the COVID-19 pandemic

**DOI:** 10.1192/j.eurpsy.2025.1276

**Published:** 2025-08-26

**Authors:** K. Rachubińska, A. M. Cybulska, E. Grochans, M. Stanisławska, D. Schneider-Matyka

**Affiliations:** 1 Department of Nursing, Pomeranian Medical University, Szczecin, Poland

## Abstract

**Introduction:**

The COVID-19 pandemic has been recognized as an international public health emergency.

**Objectives:**

The aim of our study was to identify contributors to nurses’ depression.

**Methods:**

This survey-based study was conducted in the Pomeranian Medical University Hospital no. 1 in Szczecin and involved 207 nurses. The following standardized research instruments were applied: the World Assumptions Scale, the Athens Insomnia Scale, the Impact of Event Scale - Revised, the Patient Health Questionnaire-9, the Generalized Anxiety Disorder, the Perceived Stress Scale, and a questionnaire of our own authorship.

**Results:**

The study showed that 72.95% of the subjects experienced severe stress, and 40.58% sufferred from insomnia. In addition, 65.7% of the respondents had anxiety symptoms of varying degrees of severity, and 62.8% had depressive symptoms of mild to severe severity. The mean score on the IES-R scale, reflecting a psychological impact of the COVID-19 pandemic, was 34.25 points.

**Table 1.** Influence of insomnia by AIS, anxiety by GAD-7, stress by PSS-10, World Assumptions Scale on the prevalence of depressive symptoms among nurses according to PHQ-9 (Model 1,2,3)

b - regression coefficient, βstand. - standardized regression coefficient, CI - confidence interval, ref - AIS reference level, Athens Insomnia Scale, GAD-7, generalized anxiety disorder; IES-R, Impact of Event Scale - Revised; PSS-10, The Perceived Stress Scale; WAS, The World Assumptions Scale

**Table:** 

Model	factor	b	β_stand._	-95% CI	+95% CI	p
Model 1	AIS (insomnia vs no insomnia)	1.571	0.250	0.155	0.344	0.000
	GAD-7 (total)	0.613	0.535	0.426	0.643	0.000
Model 2	AIS( insomnia vs no insomnia)	1.471	0.234	0.139	0.329	0.000
	GAD-7 (total)	0.600	0.524	0.416	0.632	0.000
	PSS-10 (steny)	0.402	0.101	0.001	0.200	0.047
Model 3	AIS(( insomnia vs no insomnia)	1.536	0.244	0.148	0.340	0.000
	GAD-7 (total)	0.616	0.537	0.428	0.646	0.000
	PSS-10 (steny)	0.442	0.111	0.011	0.211	0.029

**Conclusions:**

The COVID-19 pandemic affected the psychological health of medical staff, particularly through increased stress and anxiety symptoms. Anxiety levels and insomnia significantly affect the prevalence of depression among nurses.

**Disclosure of Interest:**

None Declared

